# Application of a multiplex salivary immunoassay to detect sporadic incident norovirus infections

**DOI:** 10.1038/s41598-019-56040-7

**Published:** 2019-12-20

**Authors:** Timothy J. Wade, Shannon M. Griffin, Andrey I. Egorov, Elizabeth Sams, Edward Hudgens, Swinburne Augustine, Stephanie DeFlorio-Barker, Trevor Plunkett, Alfred P. Dufour, Jennifer N. Styles, Kevin Oshima

**Affiliations:** 1 United States Environmental Protection Agency, Office of Research and Development, Research Triangle Park, North Carolina, USA; 2 United States Environmental Protection Agency, Office of Research and Development, Cincinnati, Ohio USA; 3 Oak Ridge Associated Universities, Oak Ridge, Tennessee USA; 4 University of North Carolina at Chapel Hill, Gillings School of Global Public Health, Environmental Sciences and Engineering Department, Chapel Hill, NC USA

**Keywords:** Viral infection, Epidemiology

## Abstract

Norovirus is one of the most common causes of gastroenteritis. Following infection, anti-norovirus salivary immunoglobulin G (IgG) rises steeply within 2 weeks and remains elevated for several months; this immunoconversion can serve as an indicator of infection. We used a multiplex salivary immunoassay to study norovirus infections among 483 visitors to a Lake Michigan beach in 2015. Saliva was collected on the day of the beach visit (S1); after 10–14 days (S2); and after 30–40 days (S3). Luminex microspheres were coupled to recombinant antigens of genogroup I (GI) and II (GII) noroviruses and incubated with saliva. Immunoconversion was defined as at least 4-fold increase in anti-norovirus IgG antibody response from S1 to S2 and a 3-fold increase from S1 to S3. Ten (2.1%) immunoconverted to either GI (2) or GII (8) norovirus. Among those who immunoconverted, 40% reported at least one gastrointestinal symptom and 33% reported diarrhea, compared to 15% (p = 0.06) and 8% (p = 0.04) among those who did not immunoconvert, respectively. The two participants who immunoconverted to GI norovirus both swallowed water during swimming (p = 0.08). This study demonstrated the utility of a non-invasive salivary immunoassay to detect norovirus infections and an efficient approach to study infectious agents in large cohorts.

## Introduction

Norovirus (NoV) infection is one of the most common causes of gastroenteritis, responsible for an estimated 700 million illnesses annually worldwide^1^. NoV are single-stranded RNA viruses, classified into seven genogroups (GI-GVII), with GI and GII responsible for most human infections^2^. NoVs are transmitted via the fecal-oral route by person-to-person transmission and contaminated food or water. Outbreaks have been associated with a range of settings including child care centers, nursing homes, hospitals, cruise ships, restaurants, and exposures to contaminated drinking and recreational waters^2^. Illnesses are characterized by sudden onset of vomiting and/or diarrhea within 12–48 hours of exposure lasting 2–3 days^2^. Immunity is short term, though repeated exposures may generate long-term resistance^2^.

NoVs cause waterborne infections in all regions of the world^3^. Whereas NoV infections tend to peak in the winter, waterborne outbreaks of norovirus infections are distributed more evenly throughout the year^4^, including summer outbreaks in freshwater lakes^5^. The risk of infection to swimmers likely depends on many factors including sources of contamination, their location relative to the beach, hydrologic conditions, environmental conditions (salinity, temperature), types of noroviruses present and their survival in water, rates of water ingestion by swimmers, the level of their pre-existing immunity to specific norovirus variants and degree of cross-protection against heterologous noroviruses present in the water.

Several studies have demonstrated that incident NoV infection can be detected by measuring NoV-specific salivary immunoglobulin G (IgG)^6–8^, which rises steeply within approximately 15 days following infection. In a recently published study based at a marine beach in Puerto Rico, we used a multiplex salivary immunoassay designed to detect IgG responses to two common GI and GII NoV variants and found that infection risk was over 5 times higher among swimmers who immersed their head than those who did not^9^. These infections, however, were mostly asymptomatic and were not associated with self-reported gastrointestinal symptoms.

This paper presents the results of a similarly designed study at a freshwater beach on Lake Michigan located in the State of Indiana in the United States in 2015. The goal of this study was to continue developing approaches to detect incident NoV infections using salivary biomarkers, to explore associations between NoV infections and swimming at a freshwater temperate location, and between norovirus infections and symptoms in a different population to further describe risk factors for sporadic NoV infections.

## Methods

### Health data and saliva sample collection

In 2015, we conducted a prospective study among beachgoers on Lake Michigan on summer weekends. Unaccompanied minors (under 18 years of age) or those who could not speak English or Spanish were ineligible to participate. Upon leaving the beach, participants were interviewed about water contact and other activities during their visit to the beach with parents or guardians providing information on participating children. Ten to fourteen days later, participants completed a brief questionnaire and returned it to the US Environmental Protection Agency (US EPA) by mail. The questionnaire addressed gastrointestinal and other symptoms experienced since the beach visit, other swimming exposures as well as other risk factors for gastrointestinal infections (consumption of undercooked meat, contact with animals, contact with other ill people).

Oral fluid samples were collected using an Oracol^TM^ sampler (Malvern Medical Developments, United Kingdom), which consists of a cylindrical sponge with a handle. Collection involves gentle rubbing of the gums to stimulate saliva and crevicular fluid (the exudate between the teeth and gums enriched with serum components, including serum IgG) production (hereafter called “saliva samples”) for approximately one minute or until the sponge is saturated. Three saliva samples were collected from each participant: A baseline (S1) sample upon leaving the beach, a second sample (S2) at 10–14 days and a third sample (S3) 30–40 days following the beach visit. Following the beach visit, sampling kits and shipping materials for the S2 and S3 sample collection were mailed to the participant’s home address. After collection, participants placed samples in a cooler with a frozen ice pack and arranged for return shipment to US EPA via overnight express mail. Upon delivery, samples were centrifuged to extract saliva from the sponge and to separate saliva from debris and then stored at −80 °C until analysis.

The study was approved by the Institutional Review Board for the University of North Carolina at Chapel Hill. Adults 18 years and older provided signed informed consent for themselves and for their children under 7. Children aged 7–17 provided signed informed assent along with signed parental permission. Infants under one year were excluded from saliva collection due to the presence of maternal antibodies, high rates of non-waterborne infection and lack of crevicular fluid. All methods were performed in accordance with the requirements and guidelines of the US EPA Human Research Protocol Office and the University of North Carolina Institutional Review Board.

### Analysis of saliva samples

Recombinant antigens (P particles, the P domain of the major capsid protein) from GI.1, GI.3, GII.3, GII.4 and GII.9 NoV genoptypes were obtained from Xi Jiang of Cincinnati Children’s Hospital Medical Center. These strains were selected to represent a range of GI and GII NoV responsible for human infections. Antigens were produced using an *E. coli* expression system described previously^10^. NoV antigens were coupled to distinct sets of Luminex magnetic microspheres using conditions described previously^6^ and in accordance with the Luminex xMAP Cookbook, 4^th^ edition^11^. Glutathione-S-transferase (GST), which was used as a protein purification tag for the recombinant NoV antigens, was coupled to an additional set of magnetic microspheres and used as an internal control.

NoV protein-coupled and control microspheres were added to wells of a standard round bottom polystyrene 96-well microplate (Corning, Tewksbury, MA) and incubated with saliva diluted 1:4 in PBS-1% BSA for 30 minutes. Plates were processed as described previously^12^ and according to the Luminex xMAP Cookbook^11^ indirect immunoassay protocol using 8 µg/mL of biotin-labeled affinity purified goat anti-human IgG detection antibody (KPL, Gaithersburg, MD) and 12 µg/mL streptavidin-phycoerythrin conjugate (SAPE; Invitrogen, Carlsbad, CA). Microplates were analyzed using a Luminex MAGPIX at default settings; median fluorescence intensity (MFI) of the reporter was estimated from at least 50 microspheres of each type and used in data analysis.

### Immunoconversion definition

Saliva samples with insufficient volume (<30 µL), low microsphere counts (less than 50 microspheres of any type successfully analyzed by the Luminex MAGPIX) and extreme anti-GST antibody reactivity (greater than 99^th^ percentile and less than 1^st^ percentile) were excluded from analysis.

Immunoconversion was used as a marker of NoV infection and was defined as described previously^6,7,9^: a four-fold increase in anti-NoV IgG MFI/anti-GST MFI ratio in S2 compared to S1. Because anti-NoV IgG remains elevated at least one month after infection^7,8^, the S3 anti-NoV IgG/anti-GST MFI ratio was required to be at least three-fold above the S1 anti-NoV IgG/anti-GST MFI ratio.

**Figure 1:**
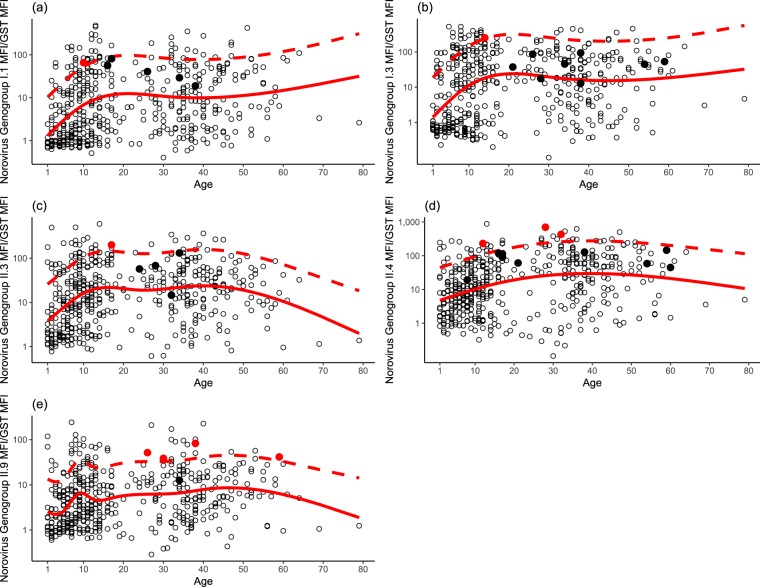
Ratio of norovirus salivary antibody response to GST at S2 sample (approximately 10–12 days after beach visit) as a function of 5-knot cubic spline of age (solid red line) and upper 75% prediction interval (dashed red line). Solid red circles represent true immunoconversions and solid black circles are immunoconversions reclassified as a non-immunoconversion due to low S2 MFI/GST MFI (see Methods). Open circles are non-immunoconversions. Panels: (a) GI.1; (b) GI.3; (c) GII.3; (d) GII.5; (e) GII.9.

To reduce potential false-positives resulting from variability in salivary IgG and the tendency for anti-NoV IgG to increase with age, the S2 anti-NoV IgG/anti-GST MFI ratio was required to be above an age-specific minimum cutoff value, as described previously^9,13^. To determine this cutoff, we modeled log_10_ transformed anti-NoV IgG/anti-GST MFI ratio as a natural cubic spline function of age and estimated the upper bound of the 90% prediction interval (Fig. 1). The degrees of freedom for the spline function ranged from one to seven with the best fitting model selected by minimizing Akaike’s Information Criterion (AIC), as recommended by Harrell^14^. To be considered an immunoconversion, the S2 anti-NoV IgG/anti-GST MFI ratio had to exceed the upper limit of the age-specific 90% prediction interval.

### Data analysis

Immunoconversion status was compared with self-reported gastrointestinal symptoms, individual and household characteristics and risk factors for gastrointestinal infection such as consumption of high-risk foods (undercooked or raw meat and shellfish), exposures to animals, swimming and other activities before, during, and after the beach visit using simple tabulations. Statistical significance was assessed using Fisher’s Exact Test. Data analysis and management was done using R software, version 3.43.

## Results

A total of 719 participants provided at least one saliva sample and completed a baseline questionnaire. Of these, 529 completed a follow up questionnaire, 484 provided an S2 sample and 370 provided all three samples. An S3 sample was only required for confirmation of immunoconversion, so those without evidence of an immunoconversion between the S1 and S2 samples did not require an S3 sample to be included in the analysis. After exclusion of samples due to extreme GST values, low volume and results with low bead count, a total of 483 participants had enough samples of sufficient volume and quality to be included in the analysis.

Antibody responses to GST normalized NoV generally increased with age up to about 10–30 years and thereafter generally flattened or declined slightly. The S2 anti-NoV/GST MFI responses for the five NoV antigens as a function of a natural cubic spline of age are shown in Fig. 1.

Table 1 shows immunoconversion status for each NoV genogroup and Table 2 lists the specific symptoms and age associated with each immunoconversion. Across all genogroups, there were 10 immunoconversions (2.1%). Eight immunoconverted to GII NoV and two to GI NoV. The most common infection was GII.9 (4, 0.8%), followed by GII.4 (3, 0.6%). There was no evidence of cross-reactivity among the NoV genogroups as all immunoconversions were associated with a single NoV.

**Table 1:** Norovirus immunoconversion frequencies.

NoV genogroup	N (%)
GI.1 (n = 480)	1 (0.2%)
GI.3 (n = 479)	1 (0.2%)
GII.3 (n = 479)	1 (0.2%)
GII.4 (n = 481)	3 (0.6%)
GII.9 (n = 480)	4 (0.8%)
GI-Any (n = 481)	2 (0.4%)
GII-Any (n = 482)	8 (1.7%)
GI or GII Any (n = 483)	10 (2.1%)

**Table 2:** Symptoms among those who immunoconverted to NoV.

Age	NoV	Diarrhea	Nausea	Vomit	Fever	Stomachache	Any gastrointestinal symptom
32	GII.4	Y	N	N	N	Y	Y
28	GII.4	NR	N	N	N	N	N
59	GII.9	Y	N	N	N	N	Y
26	GII.9	N	N	N	N	N	N
17	GII.3	N	Y	N	N	Y	Y
30	GII.9	N	N	N	N	N	N
12	GII.4	N	N	N	N	N	N
14	GI.3	N	N	N	N	N	N
10	GI.1	Y	N	N	N	N	Y
38	GII.9	N	N	N	N	N	N

Of the 10 who immunoconverted, 40% (four) reported at least one gastrointestinal symptom compared to 15% of those who did not immunoconvert (p = 0.06, Table 3). Diarrhea was most strongly associated with NoV immunoconversion with 33% (three of nine, one missing response) reporting diarrhea compared to 8% among those who did not immunoconvert (p = 0.038, Table 3). None of the participants with symptomatic immunoconversions reported that their symptoms prevented them from any regular activities.

**Table 3:** Associations between symptoms and immunoconversion status^a,b^.

	GI		GII		GI or GII	
	Yes (n = 1)	No (n = 476)	Yes (n = 8)	No (n = 474)	Yes (n = 10)	No (n = 473)
Diarrhea	1 (100%)	42 (9%)	2 (29%)	41 (9%)	3 (33%)	40 (8%)
		p = 0.09		p = 0.12		p = 0.038
Nausea	0 (0%)	30 (6%)	1 (13%)	29 (6%)	1 (10%)	29 (6%)
		p = 1		P = 0.4		p = 0.48
Stomachache	0 (0%)	55 (12%)	2 (25%)	53 (11%)	2 (20%)	53 (11%)
		p = 1		p = 0.23		p = 0.32
Vomiting	0 (0%)	13 (2.7%)	0 (0%)	13 (2.7%)	0 (0%)	13 (2.7%)
		p = 1		p = 1		p = 1
Any gastrointestinal symptom	1 (100%)	76 (16%)	3 (38%)	74 (16%)	4 (40%)	73 (15%)
		p = 0.16		P = 0.12		p = 0.059

^a^Two missing responses for diarrhea; one from an individual who immunoconverted to NoV (see Table 1).

^b^Fever was not reported among those who immunoconverted.

Comparisons of selected demographic characteristics and potential risk factors with immunoconversion status are shown in Table 4, although due to the relatively small sample size, there was low statistical power to observe associations. Those who immunoconverted to NoV were slightly but not significantly older (mean 26.6 vs. 21.0 years of age among those who did and did not immunoconvert, respectively). There was no difference in immunoconversion frequency by gender and race or potential risk factors including household size, presence of young children in the home, swimming during the baseline visit, swimming after the baseline visit, contact with other ill people, contact with unfamiliar animals, and consumption of undercooked meat (Table 4). The two participants who immunoconverted to GI NoV both reported swallowing water (1.5%) while swimming, compared to zero (0.0%) GI NoV immunoconversions among those who did not swallow water (p = 0.08).

**Table 4:** Associations between selected risk factors and demographic characteristics and immunoconversion status.

	GI	GII	GI or GII
	Yes (n = 2)	Yes (n = 8)	Yes (n = 10)
**Age**			
0–5 (n = 79)	0 (0%)	0 (0%)	0 (0%)
6–10 (n = 103)	1 (1%)	0 (0%)	1 (1%)
11–18 (n = 100)	1 (1%)	2 (2.0%)	3 (3.0%)
19–35 (n = 81)	0 (0%)	4 (5%)	4 (4.9%)
>35 (n = 120)	0 (0%)	2 (1.7%)	2 (1.7%)
	p = 0.79	p = 0.07	p = 0.21
**Sex**			
Male (n = 201)	0 (0.0%)	3 (1.5%)	3 (1.5%)
Female (n = 279)	2 (0.7%)	5 (1.8%)	7 (2.5%)
	p = 0.51	p = 0.19	p = 0.53
**Race**			
White (n = 356)	2 (0.5%)	5 (1.8%)	7 (2.0%)
Non-White (n = 111)	0 (0.0%)	2 (1.4%)	2 (1.8%)
	p = 1	p = 0.67	p = 1
**Household size**			
1–5 (n = 409)	2 (0.6%)	6 (1.5%)	8 (2.0%)
>5 (n = 74)	0 0%)	2 (2.7%)	2 (2.7%)
	p = 1	p = 0.36	p = 0.65
**Children under 5 in household**			
Yes (n = 188)	0 (0%)	5 (2.7%)	5 (1.7%)
No (n = 293)	2 (0.7%)	3 (1.0%)	5 (2.7%)
	p = 0.52	p = 0.27	p = 0.52
**Head immersion swimming during baseline beach visit**			
Yes (n = 288)	2 (0.7%)	3 (1.1%)	5 (1.7)
No (n = 195)	0 (0%)	5 (2.6%)	5 (2.6)
	p = 1	p = 0.28	p = 0.53
**Swallowed water while swimming during baseline beach visit**			
Yes (n = 137)	2 (1.5%)^a^	1 (0.74%)	3 (2.2%)
No (n = 327)	0 (0%)	5 (1.5%)	5 (1.5%)
	p = 0.08	p = 0.68	p = 0.7
**Contact with other ill people within three day prior to the baseline beach visit**			
Yes (n = 25)	1 (4.2%)	0 (1.8%)	1 (4.0%)
No (n = 458)	1 (0.2%)	8 (0%)	9 (1.9%)
	0.10	p = 1	p = 0.42
**Consumption of raw or undercooked meat**			
Yes (n = 19)	0 (0%)	1 (5.3%)	1 (5.3%)
No (n = 464)	2 (0.42%)	7 (1.5%)	9 (1.9%)
	p = 1	p = 0.28	p = 0.33

^a^100% (2/2) of GI.1 immunoconversions swallowed water, vs. 29% prevalence of swallowing water among those who did not immunoconvert to GI.1 (135/465).

## Discussion

This study confirmed that self-administered, non-invasive saliva samples collected in a community setting can be used in a multiplex, salivary immunoassay to detect symptomatic and asymptomatic infections with GI and GII NoV. A previously conducted cohort study in a community setting used a similar multiplex salivary IgG immunoassay to detect symptomatic NoV and *Cryptosporidium* sp. infections and demonstrated that measures of immunoconversion to NoV were significantly associated with gastrointestinal symptoms^13^.

During the follow up period, 2.1% (10) of participants immunoconverted to GI or GII NoV. Among those who immunoconverted, 40% (4) reported gastrointestinal symptoms, with diarrhea the most common. Vomiting, a common symptom associated with NoV infection, was not reported.

We found 4 of 483 individuals had symptomatic NoV immunoconversions, or 0.8%. From these results we can estimate the attributable risk (also referred to as the population attribution risk or attributable fraction)^15^, defined here as the fraction of symptomatic illnesses attributable to NoV infection. The attributable risk is calculated as $\frac{{I}_{T}-{I}_{o}}{{I}_{T}}\,$where I_T_ is the overall incidence in the study population and I_o_ is the incidence among those without NoV infection. This measure implies that in this study, NoV infection was responsible for 5% $(\,\frac{\frac{43}{481}-\frac{40}{472}}{\frac{43}{481}})$ of all reported diarrhea symptoms, and 3% $(\,\frac{\frac{77}{483}-\frac{73}{473}}{\frac{77}{483}})$ of all gastrointestinal symptoms. However, extrapolation of these findings to a larger population may be problematic due to the non-representative nature of the study population. Also, the true length of the follow up or risk period cannot be accurately determined because of natural variation in the timing of IgG response to NoV infection. As a result, immunoconversions may represent infections prior to and following the baseline beach visit. Finally, this multiplex immunoassay involving five norovirus antigens might not capture all norovirus infections as antibody responses to some heterologous norovirus variants could have low cross-reactivity with the assay antigens.

Although the sample size was insufficient to examine the association between NoV infection and risk factors, there was limited evidence of an association between GI NoV infection and swallowing water while swimming (100% vs. 29%, p = 0.08). GI NoVs which may be more stable in water, have been more frequently associated with waterborne and environmental transmission than GII NoVs^16^. However, this association is not conclusive as it was only of borderline statistical significance and based on just two immunoconversions which prohibited control for potential confounding factors. Moreover, we have no evidence that specific NoV genotypes were present in the beach water as we did not test water samples for NoV.

The findings of this study complement those from a similarly designed study conducted at a tropical marine beach site in Puerto Rico which reported an association between salivary IgG immunoconversion to NoV GI.1 and GII.4 and swimming exposures, but did not find an association between NoV immunoconversion and gastrointestinal symptoms^9^. In the Puerto Rico study, a slightly higher percentage of participants immunoconverted to NoV GI or GII (2.6% vs. 2.1%) and the sample size was considerably larger (n = 1298 vs. n = 483). Because of the small sample size, the present study lacked statistical power to confirm associations with swimming and other risk factors. It is unclear why NoV immunoconversion was not associated with symptoms in the Puerto Rico population, but it might have been a result of repeated exposures resulting in protection from symptoms but not infection. Errors and inconsistencies in the self-reporting of gastrointestinal symptoms may also have contributed to this observation.

This study has several limitations. Ideally, NoV infections detected via salivary IgG would be confirmed with detection of NoV in stool samples. However, the collection and shipment of stool from a study cohort is inconvenient, expensive and can have a low compliance and return rates^17^. Saliva samples are non-invasive, simple and painless to collect and can be returned via express mail without preservation. Also, we did not test water samples for the presence of NoV and therefore cannot confirm it was present at the beach. The relatively small sample size and few immunoconversions also limited our study power and precluded the ability to fully control for potential confounding factors in a multivariate analysis.

The development of this multiplex Luminex assay has been described in several previously published manuscripts^6,7,13^. In this study, the assay was used to test for IgG antibody responses to five recombinant NoV antigens representing GI.1, GI.3, GII.3, GII.4 and GII.9 genotypes. We previously demonstrated 100% sensitivity and 100% specificity for GI.1 NoVs^7^ using serial saliva samples from infected and uninfected human volunteers provided from challenge study^7^. Because of the practical, logistical and ethical difficulties of human challenge studies, they are infrequent and have not been done for every norovirus genogroup. Thus, serial saliva samples of known infected and uninfected volunteers are not available to validate the assay for each norovirus variant. Although the sensitivity and specificity of the other NoVs in this assay have not been evaluated, it has been shown that P particles of various norovirus variants have similar antigenic properties supporting the use of these recombinant antigens for measuring antibodies responses to homologous NoVs^10^. However, infections with heterologous NoVs could result in weaker observed antibody responses to the NoV antigens used in this study due to various levels of cross-reactivity. Previous research demonstrated greater cross-reactivity in antibody responses to heterologous NoV within the same genogroup than noroviruses belonging to different genogroups^7^ and very little cross-reactivity of anti-Norwalk virus (GI.1) antibody with GII.4 and GII.9 noroviruses^7^.

In conclusion, this study provides additional confirmation that a non-invasive salivary immunoassay can be used to study norovirus epidemiology and detect symptomatic and asymptomatic infections with GI and GII noroviruses. This technique provides an efficient and novel way to study infectious agents in large cohorts.

## Data Availability

This data contains personally identifiable information (PII) collected with institutional review board review and approval and an informed consent process which indicated individuals would not be identified as a result of their participation.

## References

[1] Bartsch SM, Lopman BA, Ozawa S, Hall AJ, Lee BY (2016). Global Economic Burden of Norovirus Gastroenteritis. PloS one.

[2] Glass RI, Parashar UD, Estes MK (2009). Norovirus gastroenteritis. N Engl J Med.

[3] Bitler E, Matthews J, Dickey B, Eisenberg J, Leon J (2013). Norovirus outbreaks: a systematic review of commonly implicated transmission routes and vehicles. Epidemiology & Infection.

[4] Ahmed SM, Lopman BA, Levy K (2013). A systematic review and meta-analysis of the global seasonality of norovirus. PloS one.

[5] Zlot A (2015). Norovirus outbreak associated with a natural lake used for recreation—Oregon, 2014. MMWR. Morbidity and mortality weekly report.

[6] Griffin SM, Chen IM, Fout GS, Wade TJ, Egorov AI (2011). Development of a multiplex microsphere immunoassay for the quantitation of salivary antibody responses to selected waterborne pathogens. Journal of Immunological Methods.

[7] Griffin SM (2015). Application of salivary antibody immunoassays for the detection of incident infections with Norwalk virus in a group of volunteers. Journal of Immunological Methods.

[8] Moe CL, Sair A, Lindesmith L, Estes MK, Jaykus LA (2004). Diagnosis of norwalk virus infection by indirect enzyme immunoassay detection of salivary antibodies to recombinant norwalk virus antigen. Clinical and diagnostic laboratory immunology.

[9] Wade TJ (2018). Asymptomatic norovirus infection associated with swimming at a tropical beach: A prospective cohort study. PloS one.

[10] Tan M (2011). Norovirus P particle, a novel platform for vaccine development and antibody production. Journal of virology.

[11] Angeloni, S., Das, S., Dubar, S., Stone, V. & Swift, S. xMAP® cookbook: A collection of methods and protocols for developing multiplex assays with xMAP Technology. 4th Ed., (Luminex Corp, 2018).

[12] Augustine SAJ (2017). Immunoprevalence of six waterborne pathogens in Boquerón Beach, Puerto Rico: Application of a microsphere-based salivary antibody multiplex immunoassay. Frontiers in Public Health.

[13] Egorov AI (2018). Application of a salivary immunoassay in a prospective community study of waterborne infections. Water research.

[14] Frank E. Harrell, J. *Regression Modeling Strategies*. 568 (Springer, 2010).

[15] Hennekens, C. H. & Burning, J. E. *Epidemiology in Medicine*. (Little, Brown and Company, 1987).

[16] de Graaf M, van Beek J, Koopmans MP (2016). Human norovirus transmission and evolution in a changing world. Nature Reviews Microbiology.

[17] Freedman SB (2017). Enteropathogen detection in children with diarrhoea, or vomiting, or both, comparing rectal flocked swabs with stool specimens: an outpatient cohort study. The Lancet Gastroenterology & Hepatology.

